# The Effects of Physical Activity, Exercise, and Sports Programs on Depressive Symptoms in Individuals with Disabilities: A Systematic Review with Meta-Analysis

**DOI:** 10.3390/ijerph20126134

**Published:** 2023-06-15

**Authors:** Miguel Jacinto, Diogo Monteiro, Joana Oliveira, Susana Diz, Roberta Frontini, Rui Matos, Raul Antunes

**Affiliations:** 1 School of Education and Social Sciences (ESECS), Polytechnic of Leiria, 2411-901 Leiria, Portugal; diogo.monteiro@ipleiria.pt (D.M.); joanafeoliveira@gmail.com (J.O.); rui.matos@ipleiria.pt (R.M.); raul.antunes@ipleiria.pt (R.A.); 2 Faculty of Sport Sciences and Physical Education, University of Coimbra, 3040-248 Coimbra, Portugal; 3 Life Quality Research Centre (CIEQV), 2040-413 Leiria, Portugal; roberta_frontini@hotmail.com; 4 Research Center in Sport Sciences, Health Sciences and Human Development (CIDESD), University of Trás-os-Montes and Alto Douro, 5001-801 Vila Real, Portugal; 5 Department of Sport Sciences, University of Beira Interior, 6201-001 Covilhã, Portugal; sucris.diz@gmail.com; 6 Center for Innovative Care and Health Technology (ciTechCare), Polytechnic of Leiria, 2411-901 Leiria, Portugal

**Keywords:** adapted sports, disability, mental health, physical exercise program

## Abstract

Studies show that physical activity, exercise, or sport reduces depressive symptoms in the general population. However, little is known about its effects on individuals with disabilities. Thus, this systematic review with meta-analysis aims to verify the effects of this practice on depressive symptoms in individuals with disabilities. The Pubmed, Web of Science, Scopus, and SportDiscus databases were used, with several descriptors and Boolean operators. A total of 1509 studies were identified through searching the databases. Studies that met the eligibility criteria were subsequently assessed for their methodological quality (Downs and Black scale), and a meta-analysis was performed. The *Z*-values that were obtained to test the null hypothesis, which states that there is no difference in means, showed *Z* = −2.294 and a corresponding *p*-value = 0.022. We can, therefore, reject the null hypothesis in the sense that exercise seems to reduce depressive symptoms in individuals with disabilities. In sum, participants from the intervention group presented more probability of reducing depressive symptoms when compared to the control group (approximately −1.4 standard differences in means; 95% CI −2.602 to −0.204).

## 1. Introduction

In the eleventh version of the International Statistical Classification of Diseases and Related Health Problems (ICD-11) of the World Health Organization (WHO), depression disorders are classified into the group of mental and behavioral disorders, specifically mood disorders [1].

According to the WHO [2] and the American Psychiatric Association [3], depression is a common mood disorder characterized by sadness, loss of interest or pleasure, feelings of guilt or low self-worth, disturbed sleep or appetite, tiredness, and poor concentration. Many people have lived and continue to live with depression (a report by the Institute of Health Metrics and Evaluation [4] estimated that 5% of adults suffer from the disorder), which is one of the most prevalent mental disorders in the general population [5].

In the general population, various interventions can be implemented to treat depression, including psychological and psychosocial treatments as well as antidepressant medications [2]. Recently, there has been a growing desire for self-help treatments as alternatives to medical interventions, such as pharmacological treatments [6]. Although physical activity seems to be a promising, acceptable, and recommended intervention for the treatment of adolescents and young adults with a diagnosis of depression, as recommended by NICE guidelines [7], including the supervised aerobic activity of moderate to vigorous intensity, the characteristics of the interventions still seem unclear, and the studies have some biases [8]. In the general population, individuals who do not practice physical activity are twice as likely to present symptoms of mental illness than those who do practice it [9]. In the same sense, individuals who practice physical activity with greater volume or intensity or perform strength exercises have fewer depressive symptoms [10] and suicidal thoughts [11], which highlights the importance of practice in maintaining good mental health. At the same time, some meta-analyses have confirmed that exercise is an effective treatment for depressive symptoms, with an effect size ranging from low (−0.4) [12] to high (−1.4) [13,14]. Furthermore, the effects are comparable to other first-line treatments for depression [13]. Despite limited results and studies, several systematic reviews seem to play an important role in the prevention of depressive symptomatology, but also as a treatment [13,14,15].

Having an associated disability, in its various types, is a high-risk factor for developing mental health conditions, including depression or anxiety [10,16]. The term disability encompasses all individuals with participation limitations and restrictions defined by WHO relying on the International Classification of Disease [1], namely of hearing, visual, motor, and/or intellectual, which sometimes deprive the individual of daily activities independently.

Studies report that the incidence of depressive symptoms in individuals with disabilities reaches higher numbers than in the non-disabled population [17]. On the other hand, the risk of depressive symptomatology is negatively affected by factors such as disability type and severity, being female, older age, and physical inactivity [17,18]. Furthermore, higher levels of depression are associated with poorer quality of life [19] and a higher number of suicidal thoughts and attempts [20].

In individuals with disabilities, some interventions are effective in reducing depression symptoms, namely emotional regulation training [21] and cognitive behavioral therapy [22]. In a study conducted by McGillivray and Kershaw [23], post-intervention reduction in depression scores was evident in participants of the three intervention groups: (i) cognitive strategies; (ii) behavioral strategies; and (iii) combined cognitive behavioral strategies. Nevertheless, the investigation has not specifically focused on the importance of regular physical activity, exercise, or sport as a protective element against depressive symptoms in this population.

As in the population without disabilities, for the treatment of depressive symptoms in individuals with disabilities, antidepressants are often prescribed, which in addition to being expensive, may cause side effects, including physical pain, insomnia, anxiety, fatigue, weight gain, neurological damage, motor changes, and other physiological problems [24,25], and the cost–benefits are unclear for all domains of the individuals quality of life.

Although there are several studies on depression in a population with disabilities, research on the role of physical activity, exercise, or sports in this population as a preventive factor or as a treatment of depressive symptoms is still scarce. In addition to being used for the prevention and treatment of some cardiovascular and/or metabolic diseases [26], physical activity (depending on the dose) has the potential to reduce the risk of mental health problems [27,28], which can result in reduced health care costs [29].

There is little knowledge about its effects on specific subgroups, namely individuals with disabilities, to which physical activity seems to be longitudinally associated with a reduction of depressive symptoms [30]. On the other hand, although several studies have been devoted to the analysis of the impact of exercise on the mental health of the population with disabilities (e.g., anxiety symptoms [31]), none have focused on the effects on depressive symptoms. Therefore, it is necessary to investigate the real impacts of exercise on the reduction or treatment of depressive symptoms in individuals with disabilities. Likewise, it is also important to understand the type of physical activity and the effective amounts and intensity. Therefore, this systematic review with meta-analysis aims to verify the effects of sports practice, namely physical activity, exercise, or sport on depressive symptoms in individuals with disabilities and discuss which is the most effective prescription.

## 2. Materials and Methods

### 2.1. Eligibility Criteria

The present systematic review with meta-analysis was conducted following the items of the PRISMA protocol [32] and the recommendations of the Cochrane Handbook for Systematic Reviews of Interventions, Version 6.3, 2022 [33]. The protocol of this systematic review was registered in the PROSPERO International Prospective Register of Systematic Reviews, with registration number CRD42021256218.

The PICOS strategy [34,35] was defined as follows: (i) “P” (Patients) corresponded to participants with any type of disability, of any age group, gender, ethnicity, or race; (ii) “I” (Intervention) corresponded to an exercise, physical activity, or sports program, implemented in the aforementioned population, regardless of the time of intervention; (iii) “C” (Comparison) corresponded to the comparison pre and post-intervention or between the control group (a group that was not included in the physical activity, exercise, and sports programs) and intervention group; (iv) “O” (Outcome) corresponded to depressive symptoms as the primary or secondary variable in focus; (v) “S” (Study Design) corresponded to intervention studies, randomized controlled trials (RCTs) or non-RCTs.

### 2.2. Inclusion Criteria

For the selection of studies, the following inclusion criteria were considered: (i) intervention studies, RCTs, and non-RCTs; (ii) intervention studies with an exercise program, physical activity, and sport (according to the definition by Caspersen et al. [36]); (iii) individuals with disabilities, in the most varied types; (iv) studies with individuals of any age group, gender, race or ethnicity; (v) study assessing depressive symptoms, through validated methods; (vi) studies published in scientific journals; (vii) studies with participants with disabilities and other pathologies (e.g., cardiovascular and metabolic disease), because they also benefit from physical activity, exercise or sports.

### 2.3. Exclusion Criteria

Likewise, the following exclusion criteria were considered: (i) studies that were not published in English; (ii) participants with medical contraindications for physical activity, exercise, and sports; (iii) studies that did not describe the intervention protocol.

### 2.4. Information Sources and Research Strategies

A preliminary analysis of some studies associated with the main purpose of this systematic review was conducted to identify the most appropriate databases and keywords before starting. This was also based on a previous study by the same team [31]. The search was conducted between July and October 2022 (updated up to the 21st day) in English by searching the databases PubMed (all fields), Web of Science, Scopus e SPORTDiscus (title, abstract, and keywords), considering the maximum backward period allowed by them. The following medical subject headings (MeSH) and generic descriptors were used in order to maximize the search terms: “cerebral palsy”, “motor disability”, “motor disorder”, “physical disability”, “vision impairment”, “visual impairment”, “vision disability”, “vision disorders”, “intellectual disability”, “mental retardation”, “intellectual disabilities”, “intellectual developmental disorder”, “intellectual impairment”, “hearing impairment”, “hearing disability”, “hearing loss”, “multiple disabilities”, “para athletes”, “para-athlete”, “paralympian”, “paralympians”, “paralympic athletes”, “physical activity”, “exercise”, “sports”, “training”, “depression”, “depressive disorder”, “depressive symptom” and “negative emotion”, combined with the Boolean operator “AND” and “OR”, as shown in Table 1. All terms were defined taking into consideration the eleventh version of the International Statistical Classification of Diseases and Related Health Problems (ICD-11) and previous publications [31,37], and aimed to find as many intervention studies as possible.

### 2.5. Data Extraction Process

The search was carried out independently by two researchers and downloaded information to the ENDNOTE X7 software (Clarivate, London, UK). After completion of the process, elimination of duplicate articles and those that did not meet the eligibility criteria, the results were compared by the researchers (M.J. and J.O.). After reading the full text of the articles, in accordance with the previously defined eligibility criteria, the study sample was composed of 5 articles. One of the researchers (M.J.) downloaded the relevant information from the articles and inserted them in a table (authorship, year of publication, country, objectives, participants, type of study, assessment tools, duration/frequency, exercises and intensities, and main results).

### 2.6. Methodological Quality of Study

The Downs and Black scale [38] was used to assess the quality of each study. The Downs and Black Checklist was developed to evaluate the methodological quality of randomized and nonrandomized trials. This scale consists of 27 items, scored with “two values”, “one value” or “zero”, assessing the various parts of each article. The quality of each study was independently assessed by two investigators (M.J. and J.O.). Later, the results were compared and discussed to reach a consensus or by a third investigator (R.A.). Cohen’s kappa concordance index [39] between the two investigators was 0.888. The scale was divided into several score ranges, corresponding to quality levels: excellent (26–28), good (20–25), fair (15–19), and poor (≤14). However, since six questions (questions 11, 12, 13, 16, 22, and 27) did not apply to all studies, they were removed. After modification, the scale had a maximum of 22 points compared to the original, divided into: excellent (20–22), good (14–19), fair (9–13), and poor (≤8).

### 2.7. Data Analysis

The meta-analysis followed the Preferred Reporting Items for Systematic Review and Meta-Analysis Protocols (PRISMA-P) [40]. A meta-analysis was carried out through Comprehensive Meta-analysis Version 3.0 statistical software (Biostact, Inc., Englewood, CO, USA). The standard difference in means was calculated based on previous information from the primary manuscript, namely pre-and post-intervention means, standard deviation, and number of participants. To measure the effect size, the randomized effects model was used with a 95% confidence interval (CI), magnitude effects, and level of statistical significance (*p* < 0.05). The Chi-square, Cochran *Q* statistic, Higgin I squared (*I*^2^), and Tau square tests (*T*^2^) were assessed for heterogeneity measurements. To assess the influence of each study on the overall effect size, a sensitivity analysis was carried out using the leave-one-out method (i.e., withdrawing one study at a time and then repeating the analysis) [41,42]. The asymmetry of the funnel-shaped scatter plot [43] was used to verify homogeneity. When the graph had an inverted funnel, it was considered without publication bias [44]. To classify the magnitude of the effect size of the standard means differences, Cohen’s category was selected (*d* values between 0.2 and 0.5 reflect a small effect size; between 0.5 and 0.8, a medium effect size; greater than 0.8, a large effect size) [45]. In this meta-analysis, negative effect size value favours the exercise intervention, in the sense of reducing depressive symptoms.

## 3. Results

### 3.1. Selection of Studies

**Figure 1:**
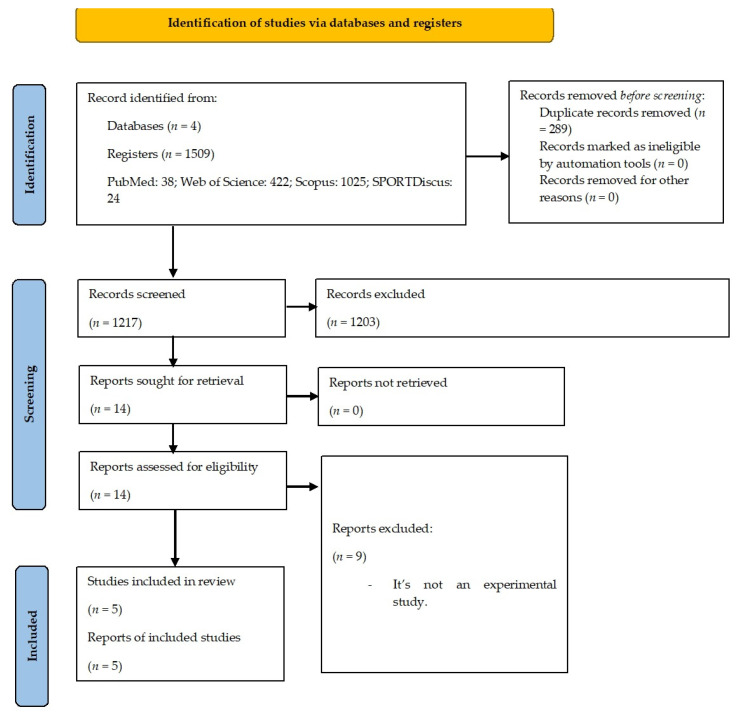
PRISMA 2020 flowchart.

A total of 1509 studies were identified through searching the databases. In the first phase, which included reading titles and abstracts, 14 studies were identified as potentially relevant for the study. Considering the previously defined eligibility criteria and after the full reading of the articles, five studies were identified as meeting the eligibility criteria and included in this systematic review for a full analysis. Figure 1 represents the flow chart of this systematic review.

### 3.2. Studies Characteristics

Table 2 presents the characteristics of the studies, namely: authors’, aims, participants, duration/frequency, exercises and intensities, measurements, results and methodology quality.

Analyzing the table of study characteristics (Table 2), the present systematic review with meta-analysis includes 226 participants, of which 111 belonged to the intervention group and 115 to the control group. All studies recruited a sample in the adult age group except for the study that recruited adolescents. Only the study by Coyle and Santiago [47] recruited a sample with physical disabilities, while the others recruited a sample with intellectual disabilities. At the same time, this was the only study not published in the last ten years and in Europe.

The studies by Coyle and Santiago [47] and Hardoy et al. [48] used a scale that assessed several variables, whereas the scales used by the other three only assessed depressive symptoms, or the authors presented the values separately. The assessment instruments were different across the various studies.

All the studies differed in terms of the intervention used. Hardoy et al. [48] and Perić et al. [49] used a sports program using mini tennis and soccer, respectively. Carraro and Gobbi [46] prescribed an intervention with exercises performed in pairs to promote physical abilities. Coyle and Santiago [47] prescribed a combined exercise program (aerobic capacity plus strength training) without further prescription in terms of volumes and intensities. Similarly, without further specifying the exercise protocol, van Schijndel-Speet et al. [50] prescribed an exercise program that focused on all physical abilities, including a dialogue session on barriers to physical activity.

No pattern was identified regarding the duration of the interventions, weekly frequency, and length of the training sessions. Still, the intervention programs lasted 12 to 24 weeks, with a weekly frequency of 2 to 4 times, and the intervention sessions lasted 45 to 180 min.

### 3.3. Methodological Quality of Studies

The quality ratings are shown in Table 2. After evaluating the methodological quality of the studies, they had a quality level ranging from good to excellent, a fact that did not lead us to exclude any studies due to low-quality scores. The studies with the highest quality were by Carraro and Gobbi [46] and Perić et al. [49], while the studies with the lowest quality assessment were developed by Coyle and Santiago [47], Hardoy et al. [48], and van Schijndel-Speet et al. [50].

### 3.4. Results of Interventions

**Figure 2:**
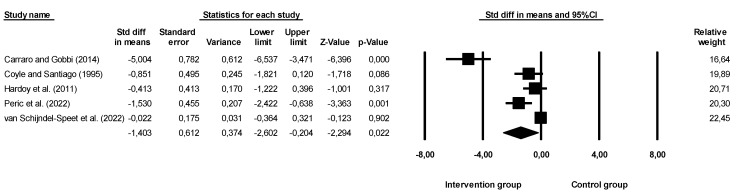
Effectiveness of interventions on depressive symptoms. Note: Carraro and Gobbi (2014), [46]; Coyle and Santiago (1995), [47]; Hardoy et al. (2011), [48]; Peric et al. (2022), [49]; van Schijndel-Speet et al. (2022), [50].

**Figure 3:**
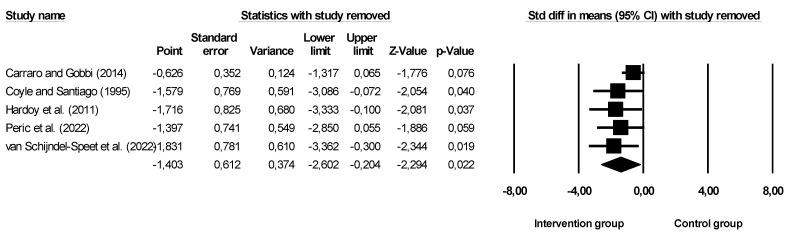
Leave-one-out sensitivity analyses for the effectiveness of the intervention on depressive symptoms. Note: Carraro and Gobbi (2014), [46]; Coyle and Santiago (1995), [47]; Hardoy et al. (2011), [48]; Peric et al. (2022), [49]; van Schijndel-Speet et al. (2022), [50].

Given the aims of this systematic review with meta-analysis, we found that not all studies that assessed depression had a decrease in these symptoms through the implementation of physical activity, exercise, or sports programs (Figure 2 and Figure 3—with sensitivity analysis).

**Figure 4:**
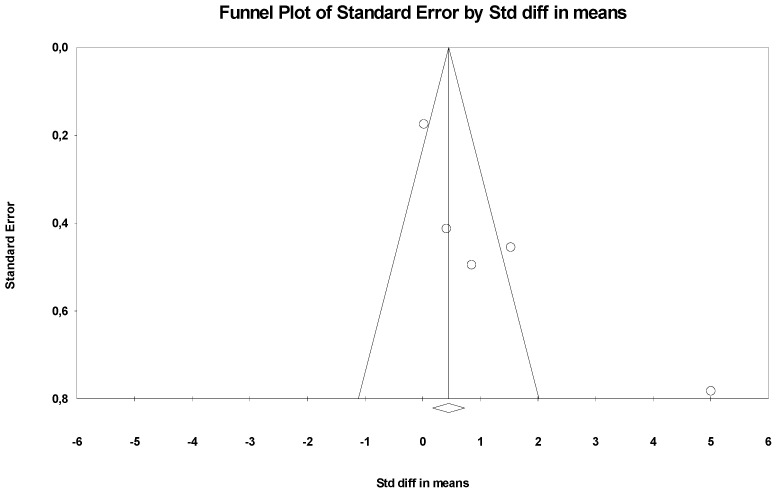
Funnel Plot.

The overall effect size was −1.403 (larger effect size), indicating that participants in the intervention group were approximately 1.4 units more likely to report a decrease when compared to the without intervention, namely the control group. Confidence intervals for standard differences of means ranged from −2.602 to −0.204. The *Z* value obtained was *Z* = −2.294, with a *p*-value of 0.022. Thus, considering that the practice of physical activity, exercise, and sport seems to affect the depressive symptoms of individuals with disabilities, the null hypothesis should be rejected. Based on the results of the sensitivity analysis, none of the studies had an impact on the overall effect, indicating that our meta-analysis was statistically stable. The obtained value of *Q* was 46.192 with six degrees of freedom and a *p*-value < 0.001, and the effect size was different in all manuscripts included in this meta-analysis. The *I*^2^ value obtained was 91.341, which means that approximately 91% of the variance in the observed effects reflected the variance of true effects. *T*^2^ showed a value of 1.637. The *T*-value showed a value equal to 1.279. Finally, the Egger test was also performed (Figure 4), which proposes to test the null hypothesis according to which the intercept is equal to zero in the population. In Figure 4, the intercept was −5.30971, and the 95% confidence interval was (−11.07041; 0.45099), with *t* = 2.933330 and *gl* = 3. The recommended *p*-value (two-tailed) was 0.06084, demonstrating no existence of publication bias.

## 4. Discussion

By analyzing the meta-analysis, we can reject the null hypothesis and state that exercise appears to promote positive effects and may help reduce depressive symptoms in individuals with disabilities. Participants in the intervention group seem to reduce depressive symptoms when compared to the control group (−1.4 standard differences in means, 95% CI −2.602 to −0.204).

Although the meta-analysis shows reductions in depressive symptoms, some of the included studies show no significant results. Several reasons can justify these individual results. In Coyle and Santiago [47], despite showing that the intervention group that completed a combined exercise program reduced depressive symptoms by 59%, the results were not significant when included in the meta-analysis. We know that the American College of Sports Medicine and Medicine [51] publishes guidelines for the assessment and prescription for the population with disability and with publication-to-publication updates, and the intervention protocol (thought to be adapted at the time) may no longer be adjusted, so the study should be taken at carefully. Similarly, a clear justification for the results not being significant in the Hardoy et al. [48] study is that the authors found no significant difference between groups. However, this author presents limitations to his study that should be taken into consideration for future experimental studies: (i) several structural limits in development and evaluation (e.g., participants aged up to 40 years old); (ii) limited resources available (without specifying); (iii) the small number of participants (*n* = 24); (iv) small frequency of sessions (without quantifying). In the same direction as the previously mentioned study, the study by van Schijndel-Speet et al. [50] showed no significant statistical value in the meta-analysis, and the authors also found no significant differences with the multidisciplinary intervention used. However, it is the study with the highest weighting in the meta-analysis, which can be justified by the high number of participants it recruited. The authors also present some limitations in their study, which are important for future studies: (i) many missing data; (ii) the approach used to fill in the missing data was not the most adequate (without specifying); (iii) despite being an RCT, none of the participants was blind for the interventions/assessments.

Despite the study of Carraro and Gobbi [46] being the study with the low weight in the meta-analysis, it is the study with the most significant results and better methodological quality (21—good). The study gives us indications that its reproducibility, namely the prescription of group activities and games (combination of physical exercise with socialization and interaction), reduces depressive symptoms in individuals with intellectual disabilities. A study with the second highest weight of the meta-analysis and with superior methodological quality is the study of Perić et al. [49]. The authors concluded that a soccer sports program had been shown to significantly reduce depressive symptoms in participants with Down syndrome. Sports activities with socialization and peer interaction (including a competitive aspect) were the ones that resulted in significant values for the meta-analysis, contrary to the general population, where supervised aerobic-based activity of moderate-to-vigorous intensity, engaged in multiple times per week, appears to be the most effective [8]. These results corroborate the study of Di Cagno et al. [52], who analyzed individuals with visual impairment who practiced and did not practice sports and found an association between this variable and well-being, namely depressive symptoms. In motor disabilities, the practice of sports reduces depressive symptoms [53]. Participation in sports activities has been considered a key element for improving well-being (namely in the treatment of depression), quality of life, breaking down social barriers of discrimination towards people with disabilities, and may translate into a reduction of health care costs [54]; thus, future research should continue to implement this methodology.

When conducting a meta-analysis to examine the effect of interventions to improve mental health problems, including depression in individuals with disabilities, Koslowski et al. [55] found a moderate and non-significant effect size for interventions with psychotherapy [*d* = 0.49, 95% confidence interval (CI) −0.05 a 1.03; *p* = 0.08)]. On the other hand, another meta-subgroup analysis indicated that for individuals with intellectual disability, individual psychological therapy (*g* = 0.778, 95% CI 0.110 to 1.445) was more effective than group psychological therapy (*g* = 0.558, 95% CI 0.212 to 0.903) and psychological interventions for depression had a moderate effect size (*g* = 0.742, 95% CI 0.116 to 1.599) [56]. The results of the present systematic review with meta-analysis indicate that physical activity, exercise or sport may be as or more effective in reducing depressive symptomatology as treatments with psychological and/or cognitive therapies.

Although the mechanisms linking sports practices to decrease depressive symptoms are still unclear [57], some biological evidence links neuromuscular mechanisms, namely increased expression of neurotrophic factors (i.e., BDNF) [58], increased serotonin and norepinephrine availability [59], regulation of HPA-axis activity [60], and decrease systemic inflammatory signaling [61], to mood and depression.

With an increased interest by various stakeholders in promoting the mental health of individuals with disabilities, our results seem to indicate that the practice of physical activity, exercise, or sports may also be a good adjuvant for reducing depressive symptoms in individuals with disabilities, with a high effect size of the interventions. However, individuals with disabilities continue to show higher rates of sedentary behavior and low adherence to physical activity, exercise, or sports compared to individuals without disabilities [62], which makes it difficult to achieve mental health benefits. Barriers to physical activity, exercise, or sports are pointed to one of the reasons for higher rates of sedentary behavior and low adherence [63,64]. The planning of strategies and interventions for this population should consider the results of this systematic review with meta-analysis, in the sense that the practice of physical activity, physical exercise, and sports, in addition to promoting physical fitness, reduces depressive symptoms and is, therefore, an asset to their quality of life.

However, the results should be considered with caution. We found only five studies that met the eligibility criteria, so the results and conclusions of this study are limited. At the same time, four of the five studies included only individuals with intellectual disabilities, one study included adolescents, and the intervention methodologies were very dichotomous, which did not allow us to perform subgroup analysis and may have influenced the results, which may not be the same for everyone [65]. This fact highlights the commitment to continue to implement physical activity, exercise, or sports programs in various types and methodologies for people with disabilities to understand the impact these different programs can have, not just in terms of physical fitness but also mental health. At the same time, no studies were found in the elderly population. The lack of intervention studies in the elderly population can be justified by biological features, namely early aging of the population with disability and premature death [66,67]. Several limitations in the primary studies or incomplete information, such as lack of information on the intervention protocol (intensity, volume, among others), made it impossible to analyze some variables. Therefore, we highlight the importance of a complete description of the intervention protocol in future studies. Future studies should also evaluate the impact of exercise on reducing depression symptoms in different gender or age groups. Although there is one study in the present meta-analysis that reports no significant value with a multidisciplinary methodology [50], further studies should investigate this relationship, i.e., not only an intervention with properly structured physical exercise but also with individualized psychological monitoring. Additionally, the mechanisms involved in reducing symptoms of depression need to be further investigated to better prescribe exercise programs for people with disabilities.

## 5. Conclusions

This systematic review with meta-analysis showed that participants in any intervention program (physical activity, exercise, and sports program) have more probability of decreasing depressive symptoms when compared to the control group (namely intellectual and physical disabilities populations). Furthermore, it points out that physical activity, exercise, and sports may be good methods to promote well-being, mental health, and quality of life also in individuals with disabilities. However, experimental studies are scarce, particularly RCTs that study the relationship between variables and have limited our results and conclusions. The present work could not mention guidelines on the ideal prescription to reduce depressive symptoms in individuals with disabilities, given the heterogeneity of interventions. However, analyzing the studies included in the present meta-analysis, interventions of a social and competitive nature (the practice of sports) seem to produce greater effects, requiring further research.

## Figures and Tables

**Table 1:** Search strategy.

**Search Strategy**	(“cerebral palsy” OR “motor disability” OR “motor disorder” OR “physical disability” OR “vision impairment” OR “visual impairment” OR “vision disability” OR “vision disorders” OR “intellectual disability” OR “mental retardation” OR “intellectual disabilities” OR “intellectual developmental disorder” OR “intellectual impairment” OR “hearing impairment” OR “hearing disability” OR “hearing loss” OR “multiple disabilities” OR “para athletes” OR “para-athlete” OR “paralympian” OR “paralympians” OR “paralympic athletes”) AND (“physical activity” OR “exercise” OR “sports” OR “training”) AND (“depression” OR “depressive disorder” OR “depressive symptom” OR “negative emotion”)

**Table 2:** Study characteristics.

Studies	Aims	Participants	Duration/Frequency	Exercise/Intensity	Measurements	Results	Methodology Quality
Carraro and Gobbi [46], Italy	Effects of exercise intervention for depressive symptoms.	*n* = 27 (♂:16); 40.1 ± 6.2 y; IDD. Randomized groups: intervention group (*n* = 14) and control group (*n* = 13).	12 weeks; 2 × week; 60 min/session.	Individual, paired, and group movement situations using small sports equipment (e.g., balls, ropes, and dumbbells), group cooperative situations, and adapted games.	The modified 19-item version of the Zung Self-Rating Depression Scale.	Intervention group (pre vs. post): 32.36 ± 1.5 vs. 23.71 ± 0.91. Control group (pre vs. post): 32 ± 1.73 vs. 29.77 ± 2.01.	21 (excellent)
Coyle and Santiago [47], United State of America	Effects of aerobic exercise on fitness and psychological health.	*n* =19 Physical disabilities. Nonrandomized groups: intervention group (*n* = 7, 35 to 57 y) and control group (*n* = 12, 26 to 52 y).	10–12 weeks; 2–4 × week; 20–60 min/session.	67–82% HRmax; Stationary cycle or an aerobic exercise videotape and continuous upper body and/or lower body movement.	Center for Epidemiological Studies Depression Scale.	Intervention group (pre vs. post): 4.71 ± 4.11 vs. 2.14 ± 3.58. Control group (pre vs. post): 3.42 ± 3.2 vs. 3.17 ± 3.38.	15 (good)
Hardoy et al. [48], Italy	Evaluating the efficacy of mini tennis programme as a therapeutic aid in the psychosocial rehabilitation of IDD.	*n* = 24; 18 to 40 y; IDD. Nonrandomized groups: intervention group (*n* = 12, 27.6 ± 6.7 y) and control group (*n* = 12, 26.9 ± 10.2).	24 weeks; 2 × week; 180 min/session.	Mini-tennis divided into 2 phases: (i): exercises to familiarise participants with equipment; (ii): development of coordination skills: visuo-manual, general dynamic, and temporospatial skills; (iii) learning of the basic tennis techniques.	Assessment and Information Rating Profile.	Intervention group (pre vs. post): 6.5 ± 4.3 vs. 5.3 ± 3.7. Control group (pre vs. post): 5.8 ± 3.7 vs. 5.9 ± 4.3.	15 (good)
Perić et al. [49], Serbia	Effects of adapted soccer on the motor learning and psychosocial characteristics.	*n* = 25 (♂); 15 to 17 y; Mean age: 15 y; Down Syndrome; Randomized groups: intervention group (*n* = 12) and control group (*n* = 13).	16 weeks; 2 × week; 45 min/session.	Soccer; 10 min warm-up (running and shuttle run with ball), 45 min of soccer-adapted training, and a 5 min of cool-down (stretching exercises).	Psychosocial variables assessment by 51 items based on previously used instruments whose metrics are available in the literature.	Intervention group (pre vs. post): 49.51 ± 10.08 vs. 43.53 ± 6.2. Control group (pre vs. post): 50.6 ± 10.47 vs. 55.91 ± 7.84.	21 (excellent)
van Schijndel-Speet et al. [50], Netherlands	Effects of physical activity programme.	*n* = 131; Down Syndrome; Randomized groups: intervention group (*n* = 66, mean age: 58.2) and control group (*n* = 65, mean age: 57.9).	24 weeks; 2 × week; 45 min/session (education sessions). 16 weeks; 24 × week; 15–45 min/session (physical activity sessions).	Discussions about experiences with physical activities and perceived barriers. PA structure to address the fitness components of muscular strength, endurance, balance, and flexibility. Feasible activities were selected and described for each of the selected components: 14 strength exercises, 18 endurance exercises, 17 balance exercises, and 6 flexibility exercises.	Dutch informant-report Signalizing Depression List.	Intervention group (pre vs. post): 27.3 ± 5.7 vs. 27.4 ± 5.6. Control group (pre vs. post): 27 ± 6.2 vs. 27 ± 6.4.	18 (good)

## Data Availability

The data presented in this study are available on request from the corresponding author.
